# Prevalence and mechanisms of aminoglycoside resistance among drug-resistant *Pseudomonas aeruginosa* clinical isolates in Iran

**DOI:** 10.1186/s12879-024-09585-6

**Published:** 2024-07-09

**Authors:** Nilofar Saeli, Saghar Jafari-Ramedani, Rashid Ramazanzadeh, Maryam Nazari, Amirhossein Sahebkar, Farzad Khademi

**Affiliations:** 1 Department of Microbiology, School of Medicine, Ardabil University of Medical Sciences, Ardabil, Iran; 2 Center for Global Health Research, Saveetha Institute of Medical and Technical Sciences, Saveetha Medical College and Hospitals, Saveetha University, Chennai, India; 3 Applied Biomedical Research Center, Mashhad University of Medical Sciences, Mashhad, Iran; 4 Biotechnology Research Center, Pharmaceutical Technology Institute, Mashhad University of Medical Sciences, Mashhad, Iran; 5 Arthropod-Borne Diseases Research Center, Ardabil University of Medical Sciences, Ardabil, Iran

**Keywords:** *Pseudomonas aeruginosa*, Aminoglycoside, Resistance

## Abstract

**Background:**

Aminoglycosides have been a cornerstone of the treatment of nosocomial infections caused by *Pseudomonas aeruginosa* for over 80 years. However, escalating emergence of resistance poses a significant challenge. Therefore, this study aimed to investigate the prevailing patterns of aminoglycoside resistance among clinical isolates of *P. aeruginosa* in Iran; as well as the underlying resistance mechanisms observed in patients referred to Ardabil hospitals.

**Methods:**

A total of 200 isolates from five hospitals were evaluated. The resistance profiles of *P. aeruginosa* isolates to tobramycin, amikacin, and netilmicin were determined using the disk diffusion method. The capacity of aminoglycoside-resistant isolates to form biofilms was assessed through a phenotypic assay, and the results were confirmed using the gene amplification technique. The presence of genes associated with aminoglycoside resistance was detected using polymerase chain reaction (PCR). Quantitative reverse transcription PCR (qRT-PCR) was performed to measure the expression levels of genes encoding the MexXY-OprM efflux pump and PhoPQ two-component system (TCS).

**Results:**

The prevalence of aminoglycoside-resistant *P. aeruginosa* isolates was 48%, with 94.7% demonstrating multidrug resistance (MDR). All aminoglycoside-resistant *P. aeruginosa* strains exhibited biofilm-forming capabilities and harbored all the genes associated with biofilm production. Among the nine genes encoding 16S rRNA methylase and aminoglycoside-modifying enzymes, three genes were detected in these isolates: *aac(6’)-Ib* (85.4%), *ant(2’’)-Ia* (18.7%), and *aph(3’)-VI* (3.1%). Additionally, all aminoglycoside-resistant *P. aeruginosa* isolates carried *mexY* and *phoP* genes, although the expression levels of *mexY* and *phoP* were 75% and 87.5%, respectively.

**Conclusion:**

Given the considerably high prevalence of aminoglycoside-resistant *P. aeruginosa* strains, urgent measures are warranted to transition towards the use of novel aminoglycosides and to uphold vigilant surveillance of resistance patterns.

**Supplementary Information:**

The online version contains supplementary material available at 10.1186/s12879-024-09585-6.

## Background

 Hospitalized patients face a heightened risk of acquiring diverse hospital-acquired infections, among which *Pseudomonas aeruginosa* stands out as a prominent causative agent, particularly in intensive care units (ICUs) [1–4]. Commonly prescribed antibiotics for combating *P. aeruginosa* infections in hospitals settings encompass β-lactams, fluoroquinolones, and aminoglycosides [5, 6]. However, effective eradication of this nosocomial pathogen from hospital environments and curbing mortality rates in severe infections present considerable challenges because of its propensity to develop resistance against a broad spectrum of antiseptics, disinfectants, and antibiotics. This has led to increased costs and prolonged hospital stay [3, 7, 8].

Aminoglycosides, known for their bactericidal properties and synergistic effects with β-lactams, are commonly employed in the treatment of various *P. aeruginosa* infections, notably pulmonary infections in patients with cystic fibrosis (CF) [8]. However, the emergence of aminoglycoside-resistant *P. aeruginosa* strains has become a global concern since its initial recognition in the 1960s [8]. The resistance of *P. aeruginosa* to aminoglycosides can be attributed to several mechanisms, including (1) alterations in outer membrane permeability facilitated by lipopolysaccharide (LPS) modifications mediated by the PhoPQ two-component system (TCS); (2) efflux systems such as MexXY-OprM; (3) ribosomal changes caused by 16S rRNA ribosomal methyltransferases; (4) production of antibiotic-inactivating enzymes such as aminoglycoside phosphotransferases (APH), aminoglycoside acetyltransferases (AAC), and aminoglycoside nucleotidyltransferases (ANT); and (5) biofilm formation [8, 9].

Previous investigations conducted in Ardabil city have underscored elevated rates of resistance and elucidated resistance mechanisms in clinical isolates of *P. aeruginosa* against penicillins, β-lactam combination agents, cephems, monobactams, carbapenems, fluoroquinolones, and lipopeptides [1, 2, 4–6, 10]. Nevertheless, comprehensive data regarding aminoglycoside-resistant strains and their underlying resistance mechanisms in this region remain elusive.

The aims of this study encompassed the investigation of the following aspects: (1) the resistance patterns of *P. aeruginosa* strains to aminoglycosides, (2) assessing the prevalence of genes encoding 16S rRNA methylase (including *rmtA*, *rmtB*, *rmtC*, *rmtD*, and *armA* genes) and aminoglycoside-modifying enzymes (such as *aac(6’)-Ib*, *aac(6’)-IIa*,* aph(3’)-VI*, and *ant(2’’)-Ia* genes), (3) evaluating both phenotypic and genotypic biofilm formation through the analysis of genes *algD*, *pslD*, *pelF*, *Ppgl*, and *PAPI-1*, and (4) investigating the expression levels of genes encoding the MexXY-OprM efflux pump (*mexY* gene) and the PhoPQ TCS (*phoP* gene) among aminoglycoside-resistant *P. aeruginosa* strains isolated from various patient specimens in Ardabil hospitals.

## Materials and methods

### Bacterial isolates

In this study, glycerol stocks of *P. aeruginosa* clinical isolates (*n* = 200) were utilized, sourced from five affiliated hospitals of Ardabil Medical University between June 2019 and May 2023. These isolates were previously identified through standard phenotypic and genotypic testing protocols. *P. aeruginosa* clinical isolates had been collected from inpatients and outpatients and duplicate isolates were excluded from the study.

### Antimicrobial susceptibility testing

The resistance profiles of *P. aeruginosa* to three aminoglycosides (i.e., tobramycin, amikacin, and netilmicin) were assessed using the disk diffusion method, following the guidelines outlined by the Clinical and Laboratory Standards Institute (CLSI, 2024) [11]. The disk diffusion procedure was conducted according to established protocols, with *P. aeruginosa* (ATCC 27,853) utilized as a control strain for consistency [4]. Colistin resistance was previously done using the colistin agar test [10].

### Biofilm formation assay

Biofilm formation ability was determined for aminoglycoside-resistance *P. aeruginosa* clinical isolates using the colorimetric microtiter plate, following the method previously described by Jabalameli et al. [12]. In this procedure, the optical density (OD) of each distinct bacterial strain was measured at 590 nm using an ELISA reader and the results were recorded as ODs (the OD of a strain) ≤ ODc (the OD of the negative control) (no biofilm producer), ODc < ODs < 2× ODc (weak biofilm producer), 2× ODc < ODs < 4× ODc (moderate biofilm producer), and 4× ODc < ODs (strong biofilm producer).

### Amplification of aminoglycosides resistance genes

The genes responsible for biofilm formation (i.e.,* algD*, *pslD*, *pelF*, *Ppgl*, and *PAPI-1* genes), 16S rRNA methylase (i.e.,* rmtA*, *rmtB*, *rmtC*, *rmtD*, and *armA* genes), aminoglycoside-modifying enzymes (i.e.,* aac(6’)-Ib*, *aac(6’)-IIa*, *aph(3’)-VI*, and *ant(2’’)-Ia* genes), efflux pump (*mexY* gene) and TCS (*phoP* gene) were detected using the polymerase chain reaction (PCR) method. Deoxyribonucleic acid (DNA) extraction from aminoglycoside-resistance *P. aeruginosa* strains was carried out using the boiling method. Each PCR reaction had a final volume of 25 µL and followed a standardized thermal cycling protocol: 1 cycle of initial denaturation at 95 °C for 5 min, followed by 30 to 34 cycles consisting of denaturation at 94 °C for 1 min, annealing at various temperatures (as specified in Table 1) for 1 min, and extension at 72 °C for 1 min. The oligonucleotide sequences of the primers used and the corresponding annealing temperatures for each gene are provided in Table 1. Sequencing of the PCR product of each amplified gene was conducted, and the sequences were utilized as positive control.

The accession numbers for these sequences are available in the NCBI GenBank repository (OR855380, OR855383 to OR855385, PP468580 to PP468582, ON920997, and OR855381).

### Expression of the MexXY-OprM efflux pump and PhoPQ TCS

The quantitative reverse transcription PCR (qRT-PCR) technique was employed to quantify the expression levels of the genes encoding MexXY-OprM efflux pump (*mexY* gene) and PhoPQ TCS (*phoP* gene). Sixteen multidrug-resistant (MDR) *P. aeruginosa* clinical isolates were selected for this analysis. These MDR isolates also were extensively drug-resistant (XDR) or pandrug-resistant (PDR) or colistin-resistant bacteria. Total RNA extraction and cDNA synthesis were conducted using TRIzol™ reagent (Bio Basic, Canada) and cDNA synthesis kit (Yekta Tajhiz Azma, Iran), respectively [13]. Each qRT-PCR reaction had a final volume of 15 µL and all reactions followed a standardized thermal cycling protocol: pre-incubation at 95 °C for 600 s, followed by 40 cycles of three amplification steps at 95 °C for 20 s, 64 °C, 62 °C, and 59 °C (for *rpsL*, *mexY*, and *phoP* genes, respectively) for 20 s, and 72 °C for 30 s. The *rpsL* housekeeping gene along with *P. aeruginosa* ATCC 27,853 were served as the reference gene and the reference strain, respectively, and changes in gene expression levels of the target genes were calculated using the comparative Ct (2^-ΔΔCt^) method. Each experiment was replicated twice, and the interpretation of the results was conducted based on previous studies [13, 14].

**Table 1:** Primers used in PCR and qRT-PCR

Gene	Oligonucleotide sequence (5′ to 3′)	Annealing temperature (°C) (PCR)	Amplicon size (bp)	Annealing temperature (°C) (qRT-PCR)	Reference
*aac(6’)-Ib*	F: TTGCGATGCTCTATGAGTGGCTA R: CTCGAATGCCTGGCGTGTTT	60	482		[15]
*aac(6’)-IIa*	F: CCATAACTCTTCGCCTCATG R: GAGTTGTTAGGCAACACCGC	61	542		[16]
*aph(3’)-VI*	F: ATGGAATTGCCCAATATTATT R: TCAATTCAATTCATCAAGTTT	51	780		[15]
*ant(2’’)-Ia*	F: GACACAACGCAGGTCACATT R: CGCATATCGCGACCTGAAAGC	60	525		[17]
*armA*	F: AGGTTGTTTCCATTTCTGAG R: TCTCTTCCATTCCCTTCTCC	53	591		[18]
*rmtA*	F: CTAGCGTCCATCCTTTCCTC R: TTTGCTTCCATGCCCTTGCC	58	635		[19]
*rmtB*	F: CCCAAACAGACCGTAGAGGC R: CTCAAACTCGGCGGGCAAGC	61	585		[20]
*rmtC*	F: GCCAAAGTACTCACAAGTGG R: CTCAGATCTGACCCAACAAG	58	752		[19]
*rmtD*	F: GAGCGAACTGAAGGAAAAAC R: CAGCACGTAAAACAGCTC	54	730		[21]
*PAPI-1*	F: CATCAACCGGATCGACGAAGT R: GTCAACCCTCTGATCCAAAAAGTT	60	462		[22]
*Pelf*	F: GAGGTCAGCTACATCCGTCG R: TCATGCAATCTCCGTGGCTT	58	789		[22]
*pslD*	F: TGTACACCGTGCTCAACGAC R: CTTCCGGCCCGATCTTCATC	60	369		[22]
*ppgL*	F: GTGGTGGGGACCTATACCGAA R: GTAGTTGGCGACGAACAGGTA	59	327		[22]
*algD*	F: CGTCTGCCGCGAGATCGGCT R: GACCTCGACGGTCTTGCGGA	63	313		[4]
*rpsL*	F: GCTGCAAAACTGCCCGCAACG R: ACCGCAGGTGTCCAGCGAACC	64	250	64	[13]
*mexY*	F: CCGCTACAACGGCTATCCCT R: AGCGGGATCGACCAGCTTTC	62	246	62	[13]
*phoP*	F: TTGCGCCACCACCTCTATAC R: GAACTGGAACGGCTTGACC	58	282	59	[This study]

## Result

In this study, a comprehensive examination of clinical strains of 200 *P. aeruginosa* revealed a prevalence rate of 48% for aminoglycoside-resistant isolates, as determined by the disk diffusion method (*n* = 96). The breakdown of resistance rates for individual aminoglycoside antibiotics was as follows: tobramycin 45.5% (91/200), amikacin 43% (86/200), and netilmicin 39.2% (33/84). Table 2 provides detailed demographic information for the 96 aminoglycoside-resistant *P. aeruginosa* isolates, including data on the hospital, specimen type, patient sex, and age.

**Table 2:** Demographic information of the 96 aminoglycoside-resistant *P. aeruginosa* isolates

Data	*n* (%)	Phenotypic resistance profiles *n* (%)	Genotypic resistance profiles *n* (%)
**Sex**			
Male	42 (43.7%)	TOB 41 (97.6%), NET 14 (33.3%), AMK 38 (90.4%)	*aac(6’)-Ib* 36 (85.7%), *ant(2’’)-Ia* 7 (16.6%), *aph(3’)-VI* 1 (2.3%)
Female	54 (56.2%)	TOB 50 (92.5%), NET 19 (35.1%), AMK 48 (88.8%)	*aac(6’)-Ib* 46 (85.1%), *ant(2’’)-Ia* 11 (20.3%), *aph(3’)-VI* 2 (3.7%)
**Age**			
0–12	2 (2%)	TOB 2 (100%), NET 2 (100%), AMK 2 (100%)	*aac(6’)-Ib* 2 (100%), *ant(2’’)-Ia* 0 (0%), *aph(3’)-VI* 0 (0%)
13–30	1 (1%)	TOB 1 (100%), NET 1 (100%), AMK 1(100%)	*aac(6’)-Ib* 1 (100%), *ant(2’’)-Ia* 1 (100%), *aph(3’)-VI* 0 (0%)
31–45	9 (9.3%)	TOB 9 (100%), NET 5 (55.5%), AMK 9 (100%)	*aac(6’)-Ib* 9 (100%), *ant(2’’)-Ia* 4 (44.4%), *aph(3’)-VI* 1 (11.1%)
46–60	29 (30.2%)	TOB 28 (96.5%), NET 9 (31%), AMK 22 (75.8%)	*aac(6’)-Ib* 23 (79.3%), *ant(2’’)-Ia* 6 (20.6%), *aph(3’)-VI* 2 (6.8%)
61–75	35 (35.3%)	TOB 31 (88.5%), NET 8 (22.8%), AMK 34 (97.1%)	*aac(6’)-Ib* 28 (80%), *ant(2’’)-Ia* 3 (8.5%), *aph(3’)-VI* 0 (0%)
> 75	20 (20.8%)	TOB 20 (100%), NET 8 (40%), AMK 17 (85%)	*aac(6’)-Ib* 19 (95%), *ant(2’’)-Ia* 4 (20%), *aph(3’)-VI* 0 (0%)
**Specimen**			
Sputum	41 (42.7%)	TOB 40 (97.5%), NET 12 (29.2%), AMK 36 (87.8%)	*aac(6’)-Ib* 35 (85.3%), *ant(2’’)-Ia* 5 (11.9%), *aph(3’)-VI* 1 (2.4%)
Urine	29 (30.2%)	TOB 26 (89.6%), NET 15 (51.7%), AMK 28 (96.5%)	*aac(6’)-Ib* 21 (72.4%), *ant(2’’)-Ia* 10 (34.4%), *aph(3’)-VI* 2 (6.6%)
Wound	17 (17.7%)	TOB 16 (95.2%), NET 3 (30.9%), AMK 14 (82.3%)	*aac(6’)-Ib* 17 (94.4%), *ant(2’’)-Ia* 2 (5.5%), *aph(3’)-VI* 0 (0%)
Blood	9 (9.3%)	TOB 9 (94.1%), NET 3 (33.3%), AMK 8 (88.8%)	*aac(6’)-*Ib 9 (83.3%), *ant(2’’)-Ia* 1 (11.1%), *aph(3’)-VI* 0 (0%)
**Hospital**			
Imam Khomeini	49 (51%)	TOB 47 (95.9%), NET 6 (12.2%), AMK 45 (91.8%)	*aac(6’)-Ib* 43 (87.7%), *ant(2’’)-Ia* 8 (16.3%), *aph(3’)-VI* 1 (2%)
Alavi	37 (38.5%)	TOB 35 (94.5%), NET 24 (64.8%), AMK 35 (94.5%)	*aac(6’)-Ib* 31 (83.7%), *ant(2’’)-Ia* 8 (21.6%), *aph(3’)-VI* 2 (5.4%)
Imam Reza	9 (9.3%)	TOB 8 (88.8%), NET 2 (22.2%), AMK 6 (66.6%)	*aac(6’)-Ib* 7 (77.7%), *ant(2’’)-Ia* 1 (11.1%), *aph(3’)-VI* 0 (0%)
Bu-Ali	1 (1%)	TOB 1 (100%), NET 1 (100%), AMK 0 (0%)	*aac(6’)-Ib* 1 (100%), *ant(2’’)-Ia* 0 (0%), *aph(3’)-VI* 0 (0%)

Furthermore, the antibiotic resistance profiles of these aminoglycoside-resistant *P. aeruginosa* isolates to a range of antibiotics were investigated. The results indicated high resistance rates, including: piperacillin 88.5% (85/96), piperacillin-tazobactam 62.5% (60/96), ceftazidime 86.4% (83/96), cefepime 91.6% (88/96), aztreonam 19.7% (19/96), imipenem 95.8% (92/96), meropenem 90.6% (87/96), ciprofloxacin 93.7% (90/96), levofloxacin 93.7% (90/96), norfloxacin 91.6% (88/96), ofloxacin 60.4% (58/96), and colistin 3.1% (3/96). Remarkably, 91 of the 96 (94.7%) aminoglycoside-resistant *P. aeruginosa* isolates exhibited multi-drug resistance (MDR).

Phenotypic and genotypic analyses demonstrated that all aminoglycoside-resistant *P. aeruginosa* isolates possessed the ability to produce biofilms. Among these isolates, 70.8% (68/96) were categorized as weak biofilm producers, 17.7% (17/96) as moderate biofilm producers, and 6.2% (6/96) as strong biofilm producers. These isolates carried all genes associated with biofilm production, including *algD*, *pslD*, *pelF*, *Ppgl*, and *PAPI-1* genes.

Based on PCR analysis, 86.4% (83/96) of the tested isolates were positive for genes encoding aminoglycoside-modifying enzymes. The most prevalent gene detected was *aac(6’)-Ib* (82/96, 85.4%), followed by *ant(2’’)-Ia* (18/96, 18.7%), and *aph(3’)-VI* (3/96, 3.1%). Notably, the *aac(6’)-IIa* gene was not identified in our study. Interestingly, among the 96 aminoglycoside-resistant *P. aeruginosa* isolates, 14 (14.5%) did not harbor any known aminoglycoside resistance genes. The genotypic and phenotypic profiles of aminoglycoside resistance among the 96 *P. aeruginosa* isolates are presented in Tables 3 and 4. Additionally, the genes responsible for methylation of the 16S rRNA in the 30 S ribosomal subunit were not found to be associated with the emergence of aminoglycoside resistance in *P. aeruginosa* isolates. Details of genotypic detection of aminoglycoside resistance-associated genes in *P. aeruginosa* clinical strains are depicted in Supplementary Figure S1.

**Table 3:** Genotypic profiles of resistance to aminoglycosides among 96 *P. aeruginosa* isolates

Profiles number	Gene combination	Gene number	Frequency *n* (%)	Isolates *n* (%)	Resistance to aminoglycosides *n* (%)
1	*aac(6’)-Ib*	1	64 (66.6%)	64 (66.6%)	TOB 62 (96.8%), NET 15 (23.4%), AMK 58 (90.6%)
2	*aph(3’)-VI*	1	0 (0%)		-
3	*ant(2’’)-Ia*	1	1 (1%)		TOB 1 (100%)
4	*aac(6’)-Ib*, *aph(3’)-VI*	2	1 (1%)	16 (16.2%)	TOB 1 (100%), NET 1 (100%), AMK 1 (100%)
5	*aac(6’)-Ib*, *ant(2’’)-Ia*	2	15 (15.6%)		TOB 14 (93.3%), NET 7 (46.6%), AMK 15 (100%)
6	*aph(3’)-VI*, *ant(2’’)-Ia*	2	0 (0%)		-
7	*aac(6’)-Ib*, *aph(3’)-VI*, *ant(2’’)-Ia*	3	2 (2%)	2 (2%)	TOB 2 (100%), NET 2 (100%), AMK 2 (100%)

**Table 4:** Phenotypic profiles of resistance to aminoglycosides among 96 *P. aeruginosa isolates*

Profiles number	Antibiotic combination	Antibiotic number (*n*)	Frequency *n* (%)	Isolates *n* (%)	Antibiotic resistance mechanisms *n* (%)
1	NET	1	1 (1%)	11 (11.4%)	*aac(6’)-Ib* 1 (100%)
2	AMK	1	3 (3.1%)		*aac(6’)-Ib* 2 (66.6%), *ant(2’’)-Ia* 1 (33.3%)
3	TOB	1	7 (7.2%)		*aac(6’)-Ib* 6 (85.7%), *ant(2’’)-Ia* 1 (14.2%)
5	TOB, AMK	2	53 (55.2%)	55 (57.2%)	*aac(6’)-Ib* 47 (88.6%), *ant(2’’)-Ia* 6 (11.3%)
7	TOB, NET	2	2 (1%)		-
9	TOB, NET, AMK	3	30 (31.2%)	30 (31.2%)	*aac(6’)-Ib* 25 (83.3%), *ant(2’’)-Ia* 10 (33.3%), *aph(3’)-VI* 3 (10%)

 The MexXY-OprM efflux pump and PhoPQ TCS genes were identified in all aminoglycoside-resistant *P. aeruginosa* isolates using the PCR analysis. However, as outlined in Table 5, the expression levels of the *mexY* and *phoP* genes, assessed via qRT-PCR, among 16 selected aminoglycoside-resistant *P. aeruginosa* isolates were 75% (12/16) and 87.5% (14/16), respectively. Details of amplification curves and melting peaks for each target in qRT-PCR are presented in Supplementary Figure S2.

**Table 5:** Characteristics of the 16 selected aminoglycoside-resistant *P. aeruginosa* isolates

Isolate number	Type of specimen	Hospital	Antibiotic resistance profiles (Phenotypic)																	Antibiotic resistance mechanisms (Genotypic)				
			Biofilm production	PIP	TZP	CAZ	FEP	ATM	IMP	MEM	TOB	AMK	NET	CIP	LVX	NOR	OFX	CST	MDR	Biofilm production	TCS overproduction	Efflux pump overproduction	Aminoglycoside-modifying enzymes	16S rRNA methylase
15	Urine	Imam Khomeini	Weak	R	R	R	R	R	R	R	R	R	R	R	R	R	R	S	+	+	**9.1**	**25.1**	-	-
24	Urine	Alavi	Weak	R	I	R	R	I	R	R	R	R	R	R	R	R	R	R	+	+	**1.8**	**6.8**	-	-
25	Urine	Alavi	Weak	R	R	R	R	R	R	R	R	R	R	R	R	R	R	S	+	+	**2.04**	3.3	*aac(6’)-Ib*, *ant(2’’)-Ia*	-
28	Urine	Alavi	Weak	R	R	R	R	R	R	R	R	R	R	R	R	R	R	S	+	+	**3.3**	**7.5**	*aac(6’)-Ib*,* aph(3’)-VI*, *ant(2’’)-Ia*	-
33	Sputum	Alavi	Weak	R	R	R	R	R	R	R	R	R	R	R	R	R	R	S	+	+	**1.1**	3.8	*aac(6’)-Ib*, *aph(3’)-VI*, *ant(2’’)-Ia*	-
34	Sputum	Alavi	Weak	R	R	R	R	R	R	R	R	R	R	R	R	R	R	S	+	+	0.3	2	*aac(6’)-Ib*	-
35	Urine	Alavi	Weak	R	R	R	R	R	R	R	R	R	R	R	R	R	R	S	+	+	**7.2**	**9.7**	*aac(6’)-Ib*, *ant(2’’)-Ia*	-
38	Sputum	Alavi	Weak	R	R	R	R	R	R	R	R	R	R	R	R	R	R	S	+	+	**3.5**	**15.1**	*aac(6’)-Ib*, *ant(2’’)-Ia*	-
40	Urine	Imam Khomeini	Weak	R	R	R	R	R	R	R	R	R	R	R	R	R	R	S	+	+	**3.4**	**10.7**	*aac(6’)-Ib*, *ant(2’’)-Ia*	-
43	Wound	Alavi	Weak	R	R	R	R	R	R	R	R	R	R	R	R	R	R	S	+	+	**10.3**	**14.5**	*aac(6’)-Ib*, *ant(2’’)-Ia*	-
97	Urine	Imam Khomeini	Moderate	R	R	R	R	R	R	R	R	R	ND	R	R	R	R	S	+	+	**6.3**	**9.5**	*aac(6’)-Ib*, *ant(2’’)-Ia*	-
103	Wound	Imam Khomeini	Strong	R	R	R	R	R	R	R	R	R	ND	R	R	R	R	S	+	+	**17.5**	**24.2**	*aac(6’)-Ib*	-
141	Sputum	Imam Reza	Moderate	I	I	R	R	I	R	R	R	S	ND	R	R	R	R	R	+	+	1	3.4	*aac(6’)-Ib*	-
165	Urine	Imam Reza	Weak	R	R	R	R	R	R	R	R	R	ND	R	R	R	R	S	+	+	**24.2**	**61.3**	*aac(6’)-Ib*	-
184	Urine	Imam Khomeini	Weak	R	R	R	R	R	R	R	R	R	ND	R	R	R	R	S	+	+	**4.7**	**9.7**	*aac(6’)-Ib*	-
197	Sputum	Imam Khomeini	Weak	R	R	R	R	R	R	R	R	R	ND	R	R	R	R	S	+	+	**3.2**	**15.5**	*aac(6’)-Ib*	-
*P. aeruginosa* ATCC 27,853																					1	1		

**Abbreviations**: PIP: piperacillin, TZP: piperacillin-tazobactam, CAZ: ceftazidime, FEP: cefepime, ATM: aztreonam, IMP: imipenem, MEM: meropenem, TOB: tobramycin, AMK: amikacin, NET: netilmicin, CIP: ciprofloxacin, LVX: levofloxacin, NOR: norfloxacin, OFX: ofloxacin, CST: colistin, R: resistant, S: susceptible, I: intermediate, ND: not determined, +: positive, -: negative. Bolded numbers represent increased gene expression

## Discussion

Since the introduction of broad-spectrum aminoglycosides in the 1940s, alongside beta-lactams and fluoroquinolones, they have remained crucial as antipseudomonal agents [13, 23]. However, a comprehensive analysis of our current and previous studies reveals a significant prevalence of aminoglycoside resistance among *P. aeruginosa* strains isolated from hospitals in Ardabil (48%), which is comparable to the resistance rates observed for beta-lactams such as penicillins (46.4% to 94%), carbapenems (33.3% to 66.7%), monobactams (42.9%), cephems (46.5% to 50%), and fluoroquinolones (52.4% to 76.2%) [4]. This is a matter of concern and underscores the potential threat posed to the health of individuals receiving medical care at Ardabil hospitals. An important contributing factor to the high levels of aminoglycoside resistance in Ardabil hospitals is their extensive use in the treatment of life-threatening infections caused by various organisms, including urinary tract infections, sepsis, and pneumonia [23]. Therefore, it is imperative to develop strategies to optimize the use of antimicrobial agents, such as combination therapy, rather than relying solely on individual agents. Among the aminoglycosides, amikacin, gentamicin, and tobramycin are the most commonly used in clinical practice [23]. Amikacin serves as an indicator antibiotic for the treatment of *P. aeruginosa* infections, and amikacin-resistant strains often display cross-resistance to other aminoglycosides [17]. In this study, among the 86 tested amikacin-resistant *P. aeruginosa* strains, resistance rates to tobramycin and netilmicin were 95.3%, and 100%, respectively. It is worth noting that the average prevalence of amikacin resistance among *P. aeruginosa* strains in Iran is 50.6% [24]. The resistance to amikacin observed in this study was higher than the rates reported in Urmia (30.7%), Hamadan (30.2%), and Zanjan (21.7%), while being lower than the rates reported in Isfahan (95.5%), Tehran (80%), Ahvaz (55.2%), and Guilan (48.8%) [24]. The prevalence of amikacin-resistant *P. aeruginosa* strains in other countries was as follows: China (85%), the USA (6%), and 11 European countries (12.9%) [25–27]. Differences in these results may be attributed to geographic variations, overuse of aminoglycosides in hospitals, self-medication practices without prescription, and differences in the overall health status of the populations studied. Amikacin and gentamicin, when combined with other antibiotic classes, are recommended for the treatment of infections caused by MDR Gram-negative organisms [23]. Gentamicin disk diffusion and MIC breakpoints for *P. aeruginosa* was deleted in new CLSI breakpoint revision [11]. Based on the previous version, the resistance rate to gentamicin observed in this study was similar to the national average (46% vs. 46.9%) [24]. Interestingly, the prevalence of MDR strains among the 96 aminoglycoside-resistant *P. aeruginosa* strains was higher than the national average (94.7%) [24]. In addition, it was higher than Ahvaz (91.9%), Hamadan (88.7%), Tehran (81.3%), Tabriz (68%), Zanjan (65%), Isfahan (63.1%), Urmia (56.9%), Guilan (45.5%), and Zahedan (16.4%) [24]. The identification of genes encoding resistance to aminoglycosides is crucial for managing drug-resistant infections and preventing treatment failure [17]. The predominant mechanism of aminoglycoside resistance involves aminoglycoside modifying enzymes [28], which was corroborated in our study, with 86.4% of the strains exhibiting this type of resistance. Among these enzymes, the *aac(6')-Ib *gene emerged as the most prevalent among aminoglycoside-resistant *P. aeruginosa* strains [28]. Our findings revealed the presence of the *aac(6')-Ib *gene in 85.4% of the strains, consistent with studies by El-Far *et al*. (94.4%) [15], Dubois *et al*. (36.5%) [16], Ahmadian *et al*. (60.4%) [29], and Jafari *et al*. (74%) [30]. The *aac(6')-Ib *gene known to confer resistance to tobramycin and amikacin [31]. In our study, 86.8% (79/91) of isolates harboring the *aac(6')-Ib *gene were resistant to tobramycin, and 88.3% (76/86) were resistant to amikacin. This underscores the significance of the *aac(6')-Ib *gene as a key determinant of tobramycin and amikacin resistance in clinical isolates of *P. aeruginosa *in Ardabil hospitals. Furthermore, other prevalent modifying enzymes identified in *P. aeruginosa* include *ant(2'')-I, aac(6')-II*, and *aph(3')-VI* genes [31]. In our investigation, we observed the presence of *ant(2'')-Ia* (18.5%) and *aph(3')-VI* (3.1%) genes among aminoglycoside-resistant *P. aeruginosa* strains, while the *aac(6')-IIa* gene was not detected. In a study conducted by Kim *et al*., the *aph(3')-VI* gene was reported as the most commonly encountered (77%) [17]. The *ant(2'')-I* and *aac(6')-II* genes are associated with resistance to gentamicin and tobramycin [31]. Based on the previous CLSI version, 94.4% of isolates harboring the *ant(2'')-Ia* gene were resistant to both gentamicin and tobramycin. The *aph(3')-VI* gene mediates resistance to amikacin [31]. However, in our analysis, no significant correlation was observed between tobramycin resistance and the presence of *aph(3')-VI* (Table 4). Considering the high prevalence of aminoglycoside resistance among *P. aeruginosa* strains in Ardabil hospitals, largely attributed to aminoglycoside-modifying enzymes, the utilization of semisynthetic aminoglycosides to overcome common resistance mechanisms is recommended [23]. One notable example of a semisynthetic aminoglycoside is plazomicin, which was approved by the FDA (Food and Drug Administration) in June 2018 for the treatment of urinary tract infections caused by certain susceptible bacteria [23]. Plazomicin was specifically engineered to circumvent aminoglycoside-modifying enzymes [23]. Fourteen aminoglycoside-resistant *P. aeruginosa* strains exhibited positive phenotypic tests but did not show the presence of genes encoding aminoglycoside-modifying enzymes. There are two possible explanations for this observation: 1) the genes encoding aminoglycoside-modifying enzymes are typically carried on plasmids [28], and 2) other resistance mechanisms, such as 16S rRNA methylases, biofilm formation, MexXY-OprM efflux pump, and TCS, may be involved. In line with a report by Kim *et al*., none of the *P. aeruginosa* strains in our study harbored the 16S rRNA methylases gene. We speculate that this is because the 16S rRNA methylases gene is encoded on the same plasmid as the aminoglycoside-modifying enzymes [28]. Decreased drug accumulation via overexpression of the MexXY-OprM efflux pump gene confers low-level intrinsic resistance to aminoglycosides in *P. aeruginosa* [28]. As shown in Table 3, aminoglycoside-resistant *P. aeruginosa* strains with resistance mechanisms independent of aminoglycoside-modifying enzymes and 16S rRNA methylase exhibited biofilm production as well as overproduction of TCS and efflux pump. Studies have indicated that extracellular DNA (eDNA), a component of the biofilm matrix, is involved in aminoglycoside resistance by inducing the expression of genes regulated by the PhoPQ TCS [9]. In the current study, 87.5% of aminoglycoside-resistant *P. aeruginosa* strains exhibited expression of the PhoPQ TCS. Some additional experiments were beyond the scope of this study and could be acknowledged as limitations but also provide opportunities for future research. These include evaluation of: 1) gene expression levels of the MexXY-OprM efflux pump and PhoPQ TCS in all aminoglycoside-resistant *P. aeruginosa* strains; 2) mutations in the PhoPQ TCS genes; and 3) the genetic relationship between bacterial strains isolated from different hospitals using a molecular typing method.

## Conclusion

Considering the high prevalence of aminoglycoside-resistant *P. aeruginosa* strains with diverse resistance mechanisms in Ardabil hospitals, the following strategies are suggested to combat bacterial resistance to aminoglycosides: 1) enhancing public awareness regarding antibiotic resistance and advocating for judicious antibiotic use, 2) tailoring antibiotic prescriptions based on local antimicrobial resistance patterns and considering combination therapy when appropriate, 3) mitigating the occurrence of hospital-acquired infections through stringent adherence to infection control protocols, 4) implementing ongoing surveillance and research initiatives to monitor the prevalence and mechanisms of aminoglycoside resistance, given its plasmid-mediated nature, and 5) transitioning towards the utilization of novel antipseudomonal antibiotics, including emerging aminoglycosides, within clinical settings.

### Supplementary Information

Supplementary Material 1

Supplementary Material 2

## Data Availability

The datasets generated and analyzed during the current study are available in the NCBI GenBank repository, under the accession numbers: OR855380, OR855383 to OR855385, PP468580 to PP468582, ON920997, and OR855381.

## Abbreviations

*P. aeruginosa*: *Pseudomonas aeruginosa*
PCR: Polymerase chain reaction
qRT-PCR: Quantitative reverse transcription PCR
TCSs: Two-component systems
MDR: Multidrug-resistant
ICUs: Intensive care units
CF: Cystic fibrosis
LPS: Lipopolysaccharide
APH: Aminoglycoside phosphotransferase
AAC: Aminoglycoside acetyltransferase
ANT: Aminoglycoside nucleotidyltransferase
CLSI: Clinical and laboratory standards institute
FDA: Food and drug administration
ATCC: American type culture collection
OD: Optical density
DNA: Deoxyribonucleic acid
RNA: Ribonucleic acid
TOB: Tobramycin
AMK: Amikacin
NET: Netilmicin
PIP: Piperacillin
TZP: Piperacillin-tazobactam
CAZ: Ceftazidime
FEP: Cefepime
ATM: Aztreonam
IMP: Imipenem
MEM: Meropenem
CIP: Ciprofloxacin
LVX: Levofloxacin
NOR: Norfloxacin
OFX: Ofloxacin
CST: Colistin
R: Resistant
S: Susceptible
I: Intermediate
