# Supplementary material for: Prevalence and mechanisms of aminoglycoside resistance among drug-resistant Pseudomonas aeruginosa clinical isolates in Iran
Source: BMC Infect Dis. 2024 Jul 9;24:680. doi: 10.1186/s12879-024-09585-6 (PMC11232330; doi:10.1186/s12879-024-09585-6)
Supplement: Supplementary file 1 — Supplementary Material 1 [file 12879_2024_9585_MOESM1_ESM.docx]

**
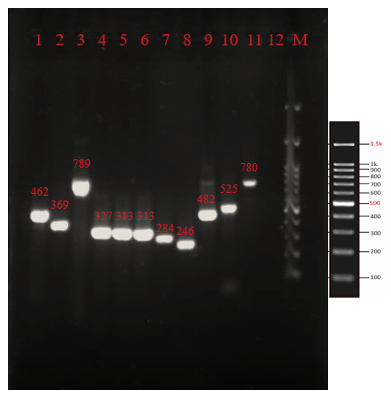
**

**Figure S1.** Agarose gel electrophoresis of amplified genes. lane 1: *PAPI-1* (462 bp), lane 2: *pslD* (369 bp), lane 3: *Pelf* (789 bp), lane 4: *ppgL* (327 bp), lanes 5 and 6: *algD* (313 bp), lane 7: *phoP* (284 bp), lane 8: *mexY* (246 bp), lane 9: *aac(6')-Ib* (482 bp), lane 10: *ant(2'')-Ia* (525 bp), lane 11: *aph(3')-VI* (780 bp), lane 12: negative control, and lane M: ladder (100 bp).
