# Supplementary material for: Prevalence and mechanisms of aminoglycoside resistance among drug-resistant Pseudomonas aeruginosa clinical isolates in Iran
Source: BMC Infect Dis. 2024 Jul 9;24:680. doi: 10.1186/s12879-024-09585-6 (PMC11232330; doi:10.1186/s12879-024-09585-6)
Supplement: Supplementary file 2 — Supplementary Material 2 [file 12879_2024_9585_MOESM2_ESM.docx]

**
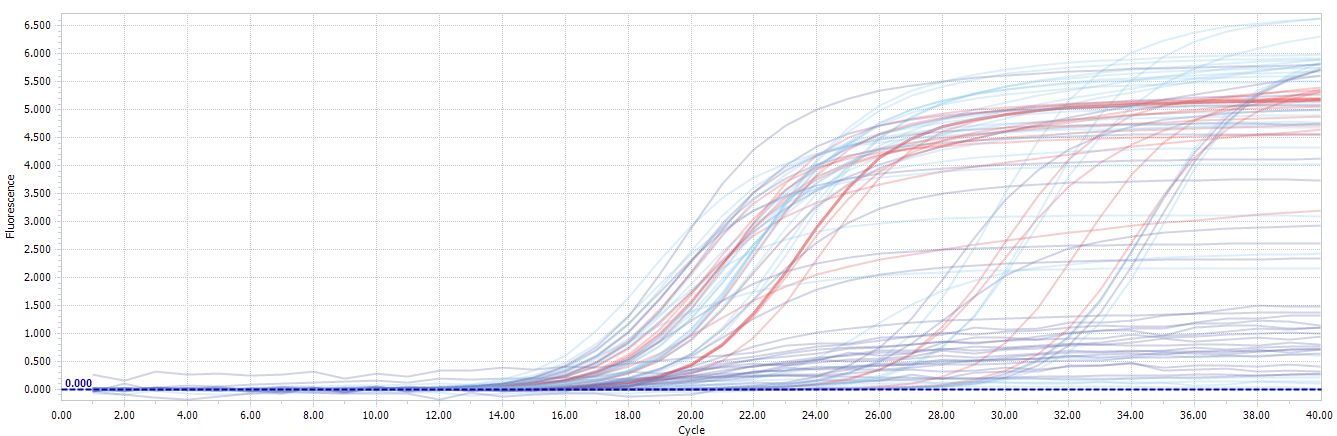
**

A

**
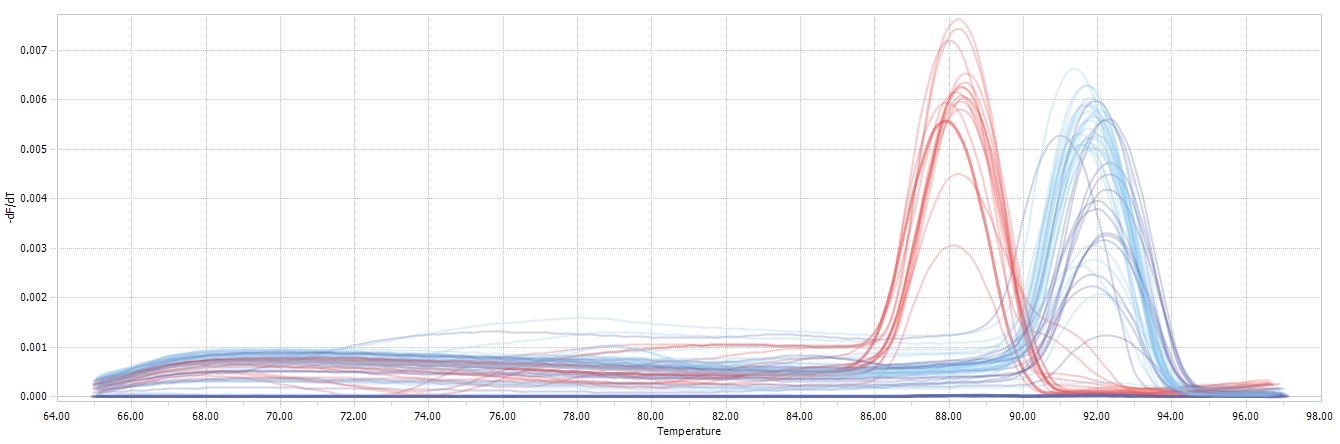
**

B

**Figure S2.** Amplification curves (A) and melting peaks (B) corresponding to for *rpsL* (reference gene, red), *mexY* (target gene, purple), and *phoP* (target gene, blue) genes.
